# NET1-mediated RhoA activation facilitates lysophosphatidic acid-induced cell migration and invasion in gastric cancer

**DOI:** 10.1038/sj.bjc.6604688

**Published:** 2008-09-30

**Authors:** D Murray, G Horgan, P MacMathuna, P Doran

**Affiliations:** 1 UCD Clinical Research Center, UCD School of Medicine and Medical Sciences, Mater Misericordiae University Hospital, 49-51 Eccles Street, Dublin 7, Ireland; 2 Gastrointestinal Unit, Mater Misericordiae University Hospital, Dublin 7, Ireland

**Keywords:** NET1, LPA, gastric cancer, invasion, migration, RhoA

## Abstract

The most lethal aspects of gastric adenocarcinoma (GA) are its invasive and metastatic properties. This aggressive phenotype remains poorly understood. We have recently identified neuroepithelial cell transforming gene 1 (NET1), a guanine exchange factor (GEF), as a novel GA-associated gene. Neuroepithelial cell transforming gene 1 expression is enhanced in GA and it is of functional importance in cell invasion. In this study, we demonstrate the activity of NET1 in driving cytoskeletal rearrangement, a key pathological mechanism in gastric tumour cell migration and invasion. Neuroepithelial cell transforming gene 1 expression was increased 10-fold in response to treatment with lysophosphatidic acid (LPA), resulting in an increase in active levels of RhoA and a 2-fold increase in cell invasion. Lysophosphatidic acid-induced cell invasion and migration were significantly inhibited using either NET1 siRNA or a RhoA inhibitor (C3 exoenzyme), thus indicating the activity of both NET1 and RhoA in gastric cancer progression. Furthermore, LPA-induced invasion and migration were also significantly reduced in the presence of cytochalasin D, an inhibitor of cytoskeletal rearrangements. Neuroepithelial cell transforming gene 1 knockdown resulted in AGS cell rounding and a loss of actin filament organisation, demonstrating the function of NET1 in actin organisation. These data highlight the importance of NET1 as a driver of tumour cell invasion, an activity mediated by RhoA activation and cytoskeletal reorganisation.

Gastric adenocarcinoma (GA) is the second most significant cause of cancer-related deaths worldwide (Hohenberger and Gretschel, 2003). Tumour metastasis is the most lethal aspect of this disease, whereby tumour cells spread to local or often distant lymph nodes. Furthermore, lymph node involvement in GA greatly affects disease prognosis and is associated with poor outcome (de Manzoni *et al*, 2001; Hohenberger and Gretschel, 2003). The invasive and metastatic abilities of these tumours and more specifically the molecular mechanisms underpinning this aggressive phenotype represent ideal targets for therapeutic intervention.

Tumour metastasis is a complex multistep process, involving detachment of cells from the primary tumour, local invasion through surrounding tissue and basement membrane, followed by intravasation into the circulatory system and ultimately extravasation and growth at distant organs. At the molecular level, tumour invasion and metastasis is dependent on the complex interplay between key biomolecules, including components of the extracellular matrix, cell adhesion molecules and proteolytic enzymes, all of which contribute to the pathological spread of tumour cells into neighbouring tissues and extracellular spaces (Poste and Fidler, 1980). Regulation of the tumour cell cytoskeleton to facilitate invasion is also an important phenotypic change in metastatic tumour cells.

The motility of tumour cells is dependent on rearrangements of the actin cytoskeleton for generating both protrusions and retraction to generate a motility cycle resulting in net translocation (Wang *et al*, 2005). Rho GTPases are an important family of proteins that regulate the actin cytoskeleton. Modulation of Rho activity promotes the metastasis of tumour cells by disrupting epithelial-sheet organisation, increasing cell motility and promoting the degradation of the extracellular matrix (Sander *et al*, 1998; Sahai and Marshall, 2002).

We have recently identified enhanced NET1 expression in GA in comparison with adjacent normal tissue and we have furthermore shown NET1 to play a role in tumour cell invasion (Leyden *et al*, 2006). Neuroepithelial cell transforming gene 1 is a member of the guanine nucleotide exchange factor (GEF) family, a group of proteins that are known to activate and thereby regulate Rho family members (Symons and Rusk, 2003; Rossman *et al*, 2005). Although the roles of other GEFs in other cancers have been established, the role of NET1 has not yet been elucidated. Other GEFs with established roles in various malignancies include; ASEF, which has been shown to promote the migration of colorectal cancer cells (Kawasaki *et al*, 2003; Nathke, 2006); Bcr, which by chromosome translocation and the formation of the Philadelphia chromosome and the BcrAbl fusion protein is essential for oncogenesis in human leukaemias (Advani and Pendergast, 2002). Another GEF, GEFH1, has been shown recently to be transcriptionally responsive to mutant p53 that resulted in increased tumour cell proliferation in a model of osteosarcoma (Mizuarai *et al*, 2006). Although NET1 was originally identified as an oncogene in neuroepithelial cells, its functional importance in other malignancies has not yet been established (Chan *et al*, 1996). Rho GTPases comprise a main branch of the Ras superfamily of small (∼21 kDa) GTPases and function as bimolecular switches, changing conformational states in response to either GDP or GTP binding. Rho proteins are inactive when bound to GDP and active when GTP-bound, actively transducing signals by interaction with downstream effector proteins (Bishop and Hall, 2000). Binding to GTP and therefore activation is promoted by Rho–GEFs and GTP hydrolysis, and therefore inactivation is catalysed by Rho–GTPase-activating proteins (Rho–GAPs). Interestingly, NET1 mRNA expression has been shown to be upregulated in response to *Helicobacter pylori* infection, an established event in gastric carcinogenesis (Chiou *et al*, 2001). In this study we aimed to further characterise the role of NET1 in GA. The effect of NET1 knockdown on RhoA activation was assessed. Furthermore, the effects of lysophosphatidic acid (LPA), a known activator of RhoA, on NET1 expression and cell motility were also investigated. The role of NET1 in LPA-induced RhoA activation and subsequent cell motility was assessed. Likewise, the role of cytoskeletal rearrangements in LPA-induced invasion and migration were investigated and the effect of NET1 knockdown on the actin cytoskeleton was also assessed. We aimed to characterise the function of NET1 in the gastric tumour setting by defining the mechanism underpinning its effect in promoting tumour cell invasion.

## Materials and methods

### Cell culture and cell treatments

AGS gastric cancer cell lines were cultured in Hams F12 medium supplemented with 10% fetal bovine serum and grown under standard conditions as previously described (Leyden *et al*, 2006). Throughout this study, cells were treated with the following: 20 *μ*M LPA for 4 h; 4 *μ*g ml^−1^ C3 exoenzyme for 4 h or 5 *μ*M cytochalasin D (CyD).

### Gene silencing by RNA interference

Neuroepithelial cell transforming gene 1 mRNA was silenced as previously described (Leyden *et al*, 2006). Briefly, siRNA duplexes were designed and synthesised for transiently silencing NET1, and a chemically synthesised non-silencing siRNA duplex that had no known homology with any mammalian gene was used to control nonspecific silencing events (Qiagen Inc., Valencia, CA, USA). The sequences were NET1 sense, 5′-GGAGGAUGCUAUAUUGAUA-3′; NET1 antisense, 5′-UAUCAAUAUAGCAUCCUCC-3′ and non-targeting sense, 5′-UUCUCCGAACGUGUCACGU-3′; antisense, 5′-ACGUGACACGUUCGGAGAA-3′. RNA interference was performed in six-well format by seeding 3 × 10^5^ cells per well. The effects of various doses of siRNA on mRNA production were investigated (0, 17, 34 and 75 nM) in a ratio of 1 *μ*g siRNA to 6 *μ*l to RNAifect (Qiagen) for 48 h. RNA and protein were extracted and analysed as described below. All RNA interference (RNAi) experiments were repeated in triplicate. Separate RNAi treatments were performed to investigate any cytotoxic effect, briefly, following RNAi treatment, viability was assessed by trypsining and counting cells using Trypan blue staining and a haemocytomoeter. To decipher any off target effects, a second NET1 siRNA duplex was used with the following sequence: sense, 5′-GGUGUGGAUUGAUUGGAAA-3′; antisense, 5′-UUUCCAAUCAAUCCACACC-3′.

### Flow cytometry

The effect of RNAi on cell viability was assessed using flow cytometry by staining with propidium iodide and Annexin V FITC. Briefly, following knockdown, cells were trypsinised and washed twice in ice-cold phosphate-buffered saline (PBS). A total of 1 × 10^5^ cells were resuspended in 100 *μ*l binding buffer (10 mM HEPES/NaOH pH7.4, 140 mM NaCl, 2.5 mM CaCl_2_) to which was added 5 *μ*l 2 mg ml^−1^ Annexin V FITC and 10 *μ*l 50 *μ*g ml^−1^ propidium iodide. Following 15 min incubation in the dark, flow cytometry was performed using a Cyan ADP analyzer (Dako, Dublin, Ireland) using 515/545 nm filter set for FITC detection and 620/640 nm set for propidium iodide. All analysis was repeated in triplicate.

### RNA extraction and PCR

TRIzol™ (Sigma-Aldrich, Dublin, Ireland) was used to extract RNA as previously described (Leyden *et al*, 2006). Following reverse transcription, as previously described (Leyden *et al*, 2006) real-time PCR was performed using a QuantiTect™ SYBR Green PCR Kit (Qiagen) following the manufacturers' instructions. Briefly, 2 *μ*l of cDNA template was mixed with 12.5 *μ*l SYBR Green master mix containing Taq and dNTPs (Qiagen), 8.5 *μ*l DNase-free water and 1 *μ*l each of forward and reverse primers. The sequences of primers used for PCR were *β*-actin forward: 5′-GTCACCTTCACCGTTCCAG-3′, reverse: 5′-CTCTTCCAGCCTTCCTTCCT-3′, NET1 forward: 5′-CTGTTCACCTCGGGACATTT-3′, reverse: 5′-TGGAGCTGTCAGACGTTTTG-3′. The reaction was carried out using a Rotor Gene™ 3000 multiplex system. All measurements were independently repeated three times. *β*-Actin mRNA expression levels were used to normalise and compare expression values for the genes of interest. The PCR products were separated on 1% agarose gels and visualised under UV light.

### Guanosine tri-phosphate-RhoA pulldown and immunoblot analysis

Guanosine tri-phosphate-bound RhoA was detected using a Rhotekin Rho-binding domain (RBD) ‘pull down’ assay (Upstate Inc., Lake Placid, NY, USA). Following NET1 knockdown, media was removed from cells before they were washed twice in ice-cold PBS and lysed in ice-cold Mg^2+^ lysis buffer (MLB) (Upstate Inc.). Cell lysates were clarified by centrifugation at 14 000 **g** for 5 min at 4°C and incubated with agarose-bound Rhotekin RBD beads (Upstate Inc.) at 4°C for 45 min. The beads were pelleted by centrifugation and washed three times in MLB before electrophoresis on a 13% SDS–PAGE gel. Bound RhoA was detected by immunoblot using an anti-Rho monoclonal antibody (Upstate Inc.). Likewise active RhoB and RhoC levels were assessed using monoclonal antibodies ab53743 and ab54837, respectively (Abcam plc, Cambridge, UK). The amount of RBD-bound Rho was normalised to the total amount of Rho in total cell lysates for the comparison of Rho activity (level of GTP-bound Rho) in different samples. Neuroepithelial cell transforming gene 1 protein was detected using a monoclonal (H70) antibody (Santa Cruz Inc., Santa Cruz, CA, USA) at a 1 : 500 dilution. *β*-Actin protein levels were detected for use as a loading control using a monoclonal antibody (Merck Biosciences, Nottingham, UK). Densitometric analysis was performed using ImageJ 1.39u software (NIH, USA). Using this software the density of the active Rho band was expressed as a ratio to the density of the total Rho band.

### Wound-healing migration assay

To investigate the migratory capacity of cells, cells were grown to 100% confluence in six-well plates in the presence of NET1-specific or scrambled siRNA. The monolayer of cells were wounded by performing a scratch with a sterile 10 *μ*l micropipette tip. Cells were washed with sterile PBS, and fresh siRNA-containing media was then added. Cells were photographed under × 10 objective lens at 24 h. Carnoy software (Biovolution) was used to measure the pixel width of the scratches.

### *In vitro* trans-well migration and invasion

Briefly, Biocoat Matrigel 8-micron invasion chambers (BD Biosciences, Bedford, MA, USA) were used to investigate the effect of cell treatments on the *in vitro* invasiveness of the AGS gastric cell line over 24 h as previously described (Leyden *et al*, 2006). Briefly, 5 × 10^5^ cells were added to Biocoat Matrigel (BD Biosciences) 24-well invasion chambers and allowed to invade for 24 h under standard culture conditions. A chemo-gradient was established by seeding cells in 1% serum and adding 20% serum to the outer chamber. Invasive cells were fixed and stained using methanol and haematoxilyn as previously described (Leyden *et al*, 2006). Invasive cells were counted in five × 10 magnification fields. Results were expressed as average cell count per field. Similarly, cell migration was monitored using 8-micron inserts without the inclusion of Matrigel.

### Fluorescent staining of cytoskeleton components

Imunofluorescent staining for F-actin assembly was carried out using rhodamine phalloidin (FAK100 kit from Chemicon International Inc., Temecula, CA, USA). Cells were grown and treated as detailed above. All growth medium was removed and cells were washed three times for 5 min with 0.05% (v/v) Tween-20 in PBS at room temperature. Cells were fixed for 15 min in 4% (w/v) p-formaldehyde in PBS at room temperature. Cells were washed as above and then permeabilised for 5 min with 0.1% Triton X-100 (v/v) in PBS at room temperature. Cells were washed and then blocked for 30 min with 1% (w/v) BSA in PBS at room temperature. Following another wash step, labelling was achieved by incubating for 1 h with 0.12 *μ*g ml^−1^ tetramethyl rhodamine isothiocyanate (TRITC)-conjugated phaloidin in PBS at room temperature in the dark. Cells were washed as above, and slides were mounted using an anitfade mounting solution (Cat no. 5013 from Chemicon International Inc.). Fluorescent images were visualised using a Zeiss Axioshop 40FL (Carl Zeiss Inc., Oberkochen, Germany).

### Statistical analysis

Data are expressed as mean±s.d. from triplicate experiments and were analysed with Microsoft Excel using a student's *t*-test with significance defined as *P*<0.05.

## Results

### The effect of NET1 on RhoA activation, gastric cancer cell migration and invasion

**Figure 1:**
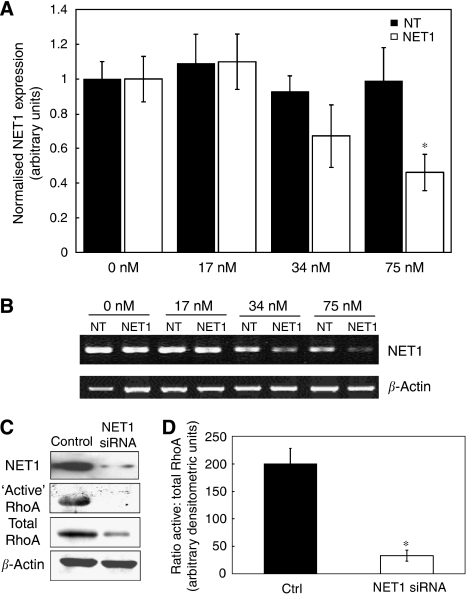
Decreased NET1 results in a reduction in the level of active RhoA. (**A**) Dose–response of NET1 mRNA expression to increasing levels of siRNA. Real-time PCR of NET1 mRNA expression in AGS cells following treatment with 0, 17, 34 or 75 nM non-target (NT) or NET1-specific siRNA in six-well format. (**B**) Agarose gel analysis of NET1 and *β*-actin PCR products in samples treated with or without increasing doses of NET1 or non-target (NT) siRNA. (**C**) Western blot analysis of control and RNAi-treated AGS cells. First panel: western immunoblot analysis of NET1 protein. Second and third panel: ‘active’ and total RhoA, respectively. Fourth panel: *β*-actin (loading control). (**D**) Ratio of active RhoA: total RhoA protein in control and NET1 siRNA samples, as determined using densitometric analysis. (^*^P<0.05). Error bars represent standard deviation of triplicate experiments.

Loss-of-function experiments were carried out to determine the role of NET1 activity in gastric cancer. An RNAi approach using an siRNA duplex specifically targeting NET1 mRNA was used to investigate the effect of NET1 knockdown on RhoA activation and AGS gastric cancer cell migration and invasion. Neuroepithelial cell transforming gene 1 mRNA production was dose–responsive to siRNA, with 75 nM siRNA causing 54% reduction in mRNA expression (*P*<0.05), whereas non-targeting siRNA caused no significant change in mRNA expression. (Figure 1A). This optimal dose was used in further experiments. The PCR products were also analysed by agarose gel electrophoresis (Figure 1B), where cells treated with 34 and 75 nM NET1 siRNA displayed decreased levels of NET1 mRNA in comparison with cells treated with the same concentration of non-targeting siRNA. Western immunoblot confirmed reduced NET1 protein levels (Figure 1C, first panel). To ensure RNAi treatments were non-cytotoxic, the viability of cells undergoing all treatments were compared using Trypan blue staining and cell counting. No significant differences were observed between groups (data not shown). Furthermore, there was no significant difference in cell viability numbers between 75 nM control and NET1 siRNA groups as measured by flow cytometry using Annexin V-FITC and propidium iodide staining (Supplementary Figure 1). Reduced NET1 mRNA resulted in an attenuation of RhoA activation (Figure 1C). The level of activated RhoA protein was dramatically reduced in response to NET1 knockdown. Neuroepithelial cell transforming gene 1 knockdown also caused a less dramatic decrease in total RhoA levels. By expressing active RhoA as a ratio against total RhoA using densitometric analysis, an 84% reduction (*P*<0.05) in this ratio was observed in cells treated with NET1 siRNA (Figure 1D). A second NET1 targeting siRNA duplex combination also resulted in decreased levels of active RhoA (Supplementary Figure 2). Knockdown of NET1 did not result in a change in the levels of active RhoB or RhoC (Supplementary Figure 3).

**Figure 2:**
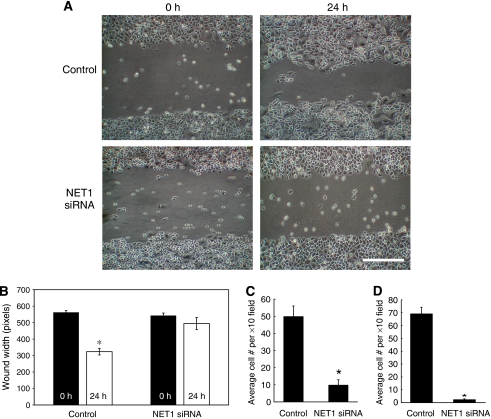
Knockdown of Net1 mRNA expression results in decreased AGS cell migration and invasion. (**A**) The effect of RNAi-mediated NET1 knockdown on AGS cell migration using a wound-healing assay in comparison with control AGS cells. Cells were treated with targeting or non-targeting siRNA for 48 h before the wound-healing assay. Cell migration was measured 24 h after the ‘wound’. Bar: 200 *μ*m. (**B**) Graphical representation of the effect of NET1 siRNA on wound healing. (**C**) The *in vitro* migratory capacity of control and NET1 knockdown cells using trans-well migration chambers. Cells were treated with targeting or non-targeting siRNA for 48 h before the migration assay, equal numbers of cells (5 × 10^4^) were added to the chambers to determine the effect of knockdown on migration over 24 h. (**D**) The *in vitro* invasive ability of control and NET1 knockdown cells using trans-well Matrigel invasion chambers. Cells were treated with targeting or non-targetting siRNA for 48 h before the migration assay, equal numbers of cells (5 × 10^4^) were added to the trans-well chambers to determine the effect of knockdown on invasion over 24 h. (^*^P<0.05). Error bars represent standard deviation of triplicate experiments.

The functional importance of NET1 in gastric cancer progression was displayed by a suppressive effect of NET1 knockdown on AGS cell migration (Figure 2A). Using a wound-healing assay, AGS cells in which NET1 knockdown was achieved were observed to be less migratory than control cells (Figure 2A). There was no significant wound healing in NET1 knockdown cells. After 24 h, control cells migrated and thereby reduced the wound width by 42% (*P*<0.005), where NET1 knockdown cells migrated and reduced the wound width 9% (Figure 2B). Using the trans-well system, 81% decreased *in vitro* migration and 94% decreased invasion was observed in AGS cells in which NET1 knockdown had been achieved (*P*<0.05) (Figure 2C and D).

### The role of RhoA activation in NET1-mediated gastric cancer cell migration and invasion

**Figure 3:**
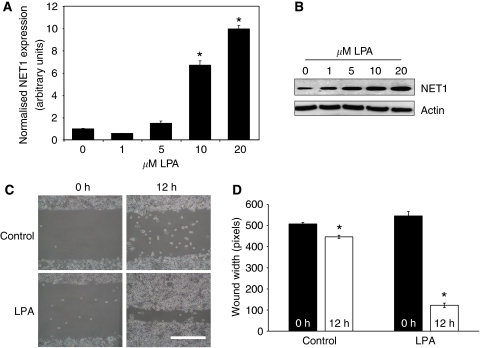
Lysophosphatidic acid (LPA) drives NET1 expression in a dose–responsive manner and promotes AGS cell migration. (**A**) Expression of NET1 mRNA in AGS gastric cancer cells treated with various doses of LPA for 4 h, as detected by quantitative real-time PCR (^*^P<0.05). (**B**) Expression of NET1 protein in AGS gastric cancer cells treated with various doses of LPA for 4 h, as detected by western immunoblot, respectively. (**C**) The effect of 20 *μ*M LPA on AGS cell migration using a wound-healing assay in comparison with control AGS cells. Cells were grown to confluence, and immediately following the ‘wound’ fresh media was added with or without LPA. Cell migration was measured 12 h after the ‘wound’. Bar, 200 *μ*m. (**D**) Graphical representation of the effect of LPA on wound healing. (^*^P<0.05). Error bars represent standard deviation of triplicate experiments.

**Figure 4:**
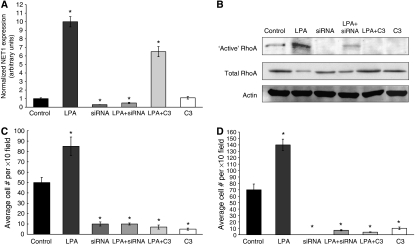
Lysophosphatidic acid (LPA)-induced NET1 expression drives AGS cell invasion by RhoA activation. (**A**) Effect of LPA, NET1 siRNA and C3 exoenzyme on NET1 mRNA expression. Cells were treated with siRNA for 48 h before treatment with or without LPA or C3 exoenzyme, 4 h after which cells were lysed and analysed by real-time PCR. Error bars represent the standard deviation of triplicate experiments. (**B**) Western blot analysis showing the effect of LPA, NET1 siRNA and C3 exoenzyme on levels of ‘active’ and total RhoA and *β*-actin. Cells were treated with siRNA for 48 h before treatment with or without LPA or C3, 4 h after which cells were lysed and analysed bya western immunoblot. All immunoblot analysis was repeated in triplicate. (**C**) The *in vitro* migration of AGS cells treated with LPA, NET1 siRNA and C3 exoenzyme. Cells were treated with siRNA for 48 h before treatment with or without LPA or C3, 24 h after which the effect on migration was assessed. Error bars represent the standard deviation of triplicate experiments. (**D**) The effect of LPA, NET1 siRNA and C3 exoenzyme on the *in vitro* invasion of AGS cells. Cells were treated with siRNA for 48 h before treatment with or without LPA or C3, 24 h after which the effect on invasion was assessed. Error bars represent the standard deviation of triplicate analysis (^*^P<0.05).

Having shown a relationship between NET1 expression and RhoA activity, the effect of LPA, a known activator of RhoA, on NET1 expression was investigated. Lysophosphatidic acid was shown to increase NET1 mRNA expression in a dose-dependent manner in AGS gastric cancer cells (Figure 3A). Likewise, treatment with LPA resulted in a dose-dependent increase in NET1 protein levels (Figure 3B). Treatment with 20 *μ*M LPA was chosen for subsequent analysis and it resulted in a 10-fold significant increase in NET1 mRNA expression (*P*<0.05) (Figure 3A). Using a 12 h wound-healing scratch assay to monitor cell migration, treatment with LPA was shown to result in 78% wound healing (*P*<0.005) in comparison with control cells in which 12% healing was achieved (*P*<0.05) (Figure 3C and D). Lysophosphatidic acid-induced expression of NET1 was blocked by siRNA-mediated knockdown of NET1 (Figure 4A). Treatment with LPA was insufficient at restoring normal levels of NET1 in cells in which NET1 knockdown had been achieved. Inhibition of RhoA activation with C3 exoenzyme did not effect LPA-driven NET1 expression and furthermore C3 exoenzyme alone had no effect on NET1 expression (Figure 4A).

The above strategy was used to delineate the activation status of RhoA in AGS cells. Treatment of AGS cells with LPA resulted in a significant increase in NET1 mRNA expression (*P*<0.05) (Figure 4A) with associated increase in the amount of active RhoA protein, while not affecting the level of total RhoA protein (Figure 4B). Lysophosphatidic acid was shown to increase the levels of active RhoA in AGS gastric cancer cells (Figure 4B). Further to our finding that NET1 knockdown resulted in a reduction in the level of active RhoA (Figure 1C), the activation of RhoA by LPA was also inhibited in cells in which RNAi-mediated knockdown of NET1 mRNA was achieved (Figure 4B). Although LPA in the presence of C3 exoenzyme resulted in increased NET1 expression, this same treatment resulted in an inhibition of RhoA activation (Figure 4B). Treatment of cells with C3 exoenzyme resulted in a complete inhibition of RhoA activation (Figure 4B).

The effects of altered NET1 and RhoA levels on tumour cell chemotaxis were examined *in vitro*. Using a 12 h wound-healing scratch assay to monitor cell migration, treatment with LPA was shown to result in 78% wound healing (*P*<0.005) in comparison with control cells in which 12% healing was achieved (*P*<0.05) (Figure 3C and D). Treatment with LPA induced a 70% increase in AGS gastric cancer cell migration and 100% increased invasion *in vitro* (*P*<0.05) (Figure 4C and D). Knockdown of NET1 significantly reduced the level of LPA-mediated chemotaxis. Lysophosphatidic acid-induced migration was reduced by 88% and LPA-induced invasion was reduced by 93% in cells in which NET1 was silenced (*P*<0.05) (Figure 4C and D). Although LPA was shown to drive the expression of NET1 in the presence of the RhoA inhibitor C3 exoenxyme (Figure 4A), these cells had 91 and 96% reduced migratory and invasive capabilities in comparison with cells treated with LPA alone (*P*<0.05) (Figure 4C and D). Treatment with C3 exoenzyme alone resulted in 90% reduced migration and 86% reduced invasion in comparison with control cells (*P*<0.05) (Figure 4C and D).

### The role of cytoskeletal remodelling events in NET1-mediated gastric cancer cell migration and invasion

**Figure 5:**
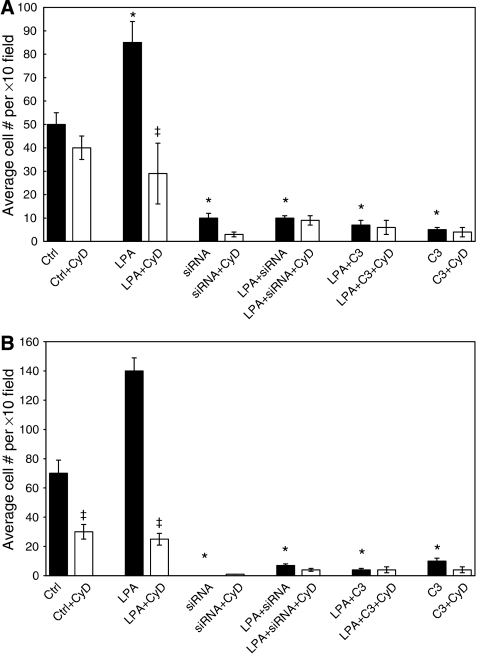
Lysophosphatidic acid (LPA)-mediated cell invasion occurs by cytoskeletal rearrangement events. The effect of cytochalasin D (CyD) on (**A**); the *in vitro* migration of AGS gastric cancer cells treated with LPA, NET1 siRNA or C3 exoenzyme, and (**B**) the *in vitro* invasion of these cells. Cells were treated with siRNA for 48 h before treatment with or without LPA, C3 or CyD, 24 h after which the effects on invasion and migration were assessed (^*^,^‡^*P*<0.05). Error bars represent standard deviation of triplicate experiments.

**Figure 6:**
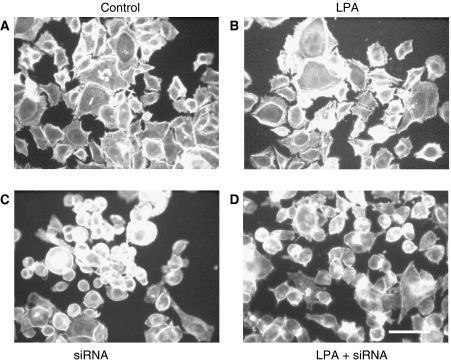
NET1 knockdown blocks the organisation of the actin cytoskeleton. Fluorescence microscopy of F-actin formation in (**A**) control AGS cells, (**B**) cells treated with LPA, (**C**) cells in which NET1 knockdown was achieved and (**D**) cells treated with LPA and NET1 siRNA. Cells were treated with siRNA for 48 h before treatment with or without LPA, 4 h after which the effect on actin polymerisation was assessed. F-actin was detected using TRITC-conjugated phalloidin (green). Nuclei were counter stained using propidium iodide (orange). Bar 40 *μ*m.

Having demonstrated a role for NET1 in mediating invasion, the effect of NET1 on cytoskeletal rearrangements was investigated. Cytochalasin D, an inhibitor of actin filament rearrangement significantly inhibited LPA-induced AGS cell migration and invasion by 67 and 84%, respectively (*P*<0.05) (Figure 5A and B). The invasion of control cells was also significantly reduced by 46% in the presence of CyD (*P*<0.05) (Figure 5B). Cytochalasin D reduced the migration of control cells, although this reduction was not statistically significant (Figure 5A). Control cells and cells treated with LPA displayed well-defined actin filament structures (Figure 6A and B). Knockdown of NET resulted in cell rounding and a loss of definition in the actin cytoskeleton (Figure 6C) in comparison with cells treated with nonspecific siRNA (Figure 5A). Cells treated with LPA in which NET1 knockdown had been achieved also appeared round with less actin cytoskeleton organisation than control cells (Figure 6D).

## Discussion

**Figure 7:**
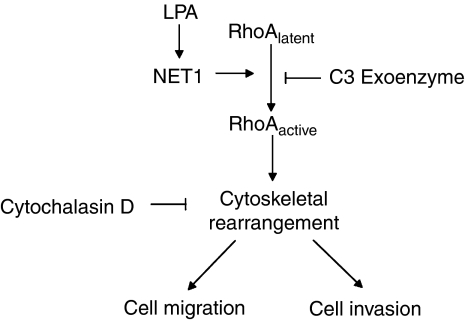
Summary of the mechanism of NET1-mediated tumour migration and invasion. In this study lysophosphatidic acid (LPA) was shown to drive the expression of NET1, which increased the activation of RhoA (a process that was inhibited by C3 exoenzyme). Knockdown of NET1 was shown to reduce the levels of active RhoA and also a loss in the organisation of the cytoskeletal architecture. Knockdown of NET1 furthermore resulted in a decrease in the levels of tumuor cell migration and invasion.

We have previously identified NET1 as being upregulated in GA and to participate in gastric cancer cell proliferation and invasion (Leyden *et al*, 2006). In this current study, our aim was to further characterise the mechanisms underpinning NET1-mediated gastric cancer cell invasion (Figure 7).

Neuroepithelial cell transforming gene 1 is a GEF and an activator of RhoA (Leyden *et al*, 2006). Rho proteins, once activated, stimulate signalling in multiple pathways by binding to downstream effector proteins, modulating their activities and thereby regulating a range of cellular processes. Rho-family proteins are regulators of the actin cytoskeleton, cell-cycle progression and gene transcription, and have been implicated in cellular processes such as adhesion and migration, cellular morphogenesis and polarisation (Hall, 1998; Evers *et al*, 2000; Sahai and Marshall, 2002; Raftopoulou and Hall, 2004).

Using an RNAi-based approach, NET1 was shown to activate RhoA with knockdown of NET1 inhibiting the activation of RhoA by 84% (Figure 1C and D). A second siRNA set was used to confirm NET1-mediated activation of RhoA, thereby ruling out off target effects (Supplementary Figure 2). Interestingly, NET1 knockdown resulted in a decrease in total RhoA levels, thereby suggesting that as well as regulating RhoA activation, NET1 may play a role in RhoA transcription. This is the first report that NET1 drives the activation of RhoA in GA. This finding strengthens the role of RhoA in gastric cancer and furthermore elaborates on the biology of NET1, a protein whose role in the disease is not yet fully understood. Interestingly, NET1 knockdown did not result in a change in the levels of either RhoB or RhoC (Supplementary Figure 3), thereby supporting its role as a RhoAspecific exchange factor in AGS cells.

Because of the established role of RhoA in cell migration and invasion (Itoh *et al*, 1999; Somlyo *et al*, 2000; Ridley, 2001), the effect of NET1 knockdown and therefore reduced levels of active RhoA, on these cellular processes in AGS cells was investigated. As expected, knockdown of NET1 resulted in a significant decrease in gastric cancer cell migration, as assed using the *in vitro* wound-healing assay (Figure 2A and B). The importance of NET1 in tumour cell chemotaxis was further demonstrated using the trans-well system, whereby AGS cells, in which NET1 knockdown has been achieved, were shown to be less migratory and invasive *in vitro* (Figure 2C and D). These data highlight the importance of RhoA biology in the gastric cancer invasive programme as well as establishing the role of NET1 in this setting.

Rho activation is frequently mediated through various cell-surface receptors, including the tyrosine kinase, cytokine and adhesion receptors and also the G-protein-coupled receptors (Kjoller and Hall, 1999; Dorsam and Gutkind, 2007). Lysophosphatidic acid is a well-established driver of RhoA activation and has been shown to drive RhoA-mediated cytoskeletal rearrangement events (Hart *et al*, 1998; Mao *et al*, 1998; Mills and Moolenaar, 2003). Lysophosphatidic acid is known to promote the migration of colorectal cancer cells and to furthermore drive their secretion of proangiogenic factors such as VEGF, which is essential for metastasis (Shida *et al*, 2003). In this study, LPA was shown to drive the expression of NET1 mRNA and protein in a dose-dependent manner (Figure 3A and B). Although the role of LPA in transcription remains to be fully understood, reports suggest that LPA-induced gene expression is mediated through peroxisome proliferator-activated receptor, a transcriptional factor identified as an intracellular LPA receptor (McIntyre *et al*, 2003). Furthermore, we have previously shown that NET1 mRNA expression is also driven by treatment with TNF*α* (Leyden *et al*, 2006). Although not explored in this study, the potential relationship between TNF*α* and LPA in driving NET1 mRNA transcription remains to be established. Indeed treatment with TNF*α* has been shown to drive the expression of LPA receptor mRNA, which is one mechanism worth exploring in the future (Zhao *et al*, 2008). As expected, treatment with LPA resulted in an increase in the levels of active RhoA (Figure 4B). Cells treated with LPA were significantly more migratory and invasive than control cells (Figures 3C, D and 4C, D). Although LPA resulted in a 10-fold increase in NET1 mRNA levels, this did not translate into a 10-fold increase in migration and invasion, suggesting that NET1 protein levels may be at a plateau in these cells. Knockdown of NET1 was shown to inhibit LPA-induced activation of RhoA (Figure 4B) and furthermore LPA-induced cell migration and invasion (Figure 4C and D). Interestingly, the level of active RhoA in these cells was similar to control cells, yet their invasion and migration was reduced, demonstrating that NET1 is not essential for LPA-mediated activation of RhoA, as well as suggesting that NET1 may mediate migration and invasion through other mechanisms besides RhoA. Inhibition of RhoA activation with C3 exoenzyme did not affect LPA-driven NET1 expression and furthermore C3 exoenzyme alone had no effect on NET1 expression (Figure 4A). Taken together, these data show that NET1 expression and activity lie upstream of RhoA biology and that LPA is driving RhoA activation through NET1. Although LPA was shown to increase the expression of NET1 in the presence of C3 exoenzyme (Figure 4A), these cells had reduced levels of migration and invasion (Figure 4C and D). As well as highlighting the importance of NET1 and RhoA in cell invasion, these data demonstrate that both NET1 and RhoA bioactivities are essential in LPA-induced gastric cancer cell migration and invasion. Furthermore, we have shown that NET1 inhibition is as potent at curbing cell migration and invasion as a RhoA inhibitor. These data firmly establish the role of enhanced NET1 expression in this disease, whereby NET1 is of key importance in gastric cancer cell invasion.

Further to our data demonstrating that LPA drives tumour cell invasion through both NET1 and RhoA, we next investigated the role of the cytoskeleton in these processes. Regulation of the cytoskeleton is a major function of RhoA activity (Hall, 1998; Matsui *et al*, 1998). Cytochalasin D was used in these studies. Cytochalasin D is a mycotoxin that inhibits both the association and dissociation of actin filament subunits by causing disruption of actin filaments and furthermore the inhibition of actin polymerisation (Goddette and Frieden, 1986). Treatment with CyD caused reduced migration and invasion of AGS cancer cells (Figure 5A and B). Lysophosphatidic acid-induced cell migration and invasion were dramatically reduced with CyD treatment (Figure 5A and B). These data support our hypothesis that LPA-induced chemotaxis of AGS gastric cancer cells occurs by cytoskeletal rearrangement events, which are mediated at least in part by NET1.

We have shown that LPA-mediated cell invasion involves increased NET1 expression and RhoA activation. To further elucidate the underlying mechanism promoting cell invasion, the effect of various treatments on the actin cytoskeleton were investigated as Rho proteins are known key mediators of the actin organisation (Van Aelst and D’Souza-Schorey, 1997; Ellis and Mellor, 2000). Control AGS cells demonstrated defined actin filament organisation (Figure 6A). Upon treatment with LPA, these filaments became more pronounced and cells appeared elongated and stretched (Figure 6B). Lysophosphatidic acid is known to mediate actin cytoskeletal changes by Rho proteins and furthermore to facilitate cell migration (Jalink *et al*, 1994; Van Leeuwen *et al*, 2003). Cells in which NET1 knockdown had been achieved failed to demonstrate the organisation of actin filaments observed in control or LPA-treated cells (Figure 6C). These cells have been shown to be less migratory and invasive than control cells (Figure 4C and D), suggesting that NET1 mediates gastric cancer cell invasion through cytoskeletal-dependent events.

This study further elaborates on the role of NET1, a novel GA-associated GEF in the disease. We have previously shown that NET1 is enhanced in gastric cancer tissue in comparison with normal tissue (Leyden *et al*, 2006) and this study further elucidates the mechanism by which NET1 mediates the progression of the disease. We have shown that NET1 is a key player in LPA-induced activation of RhoA and the subsequent migration and invasion of gastric tumour cells. NET1 inhibition was as effective at reducing cell invasion as treatment with the RhoA inhibitor, C3 exoenzyme or the inhibitor of cytoskeletal rearrangement CyD, highlighting its importance in this setting. As NET1 is important to the invasive phonotype, we therefore propose that NET1 is an ideal potential therapeutic target in this disease.
