# Supplementary material for: NET1-mediated RhoA activation facilitates lysophosphatidic acid-induced cell migration and invasion in gastric cancer
Source: Br J Cancer. 2008 Sep 30;99(8):1322–9. doi: 10.1038/sj.bjc.6604688 (PMC2570507; doi:10.1038/sj.bjc.6604688)
Supplement: Supplementary Figures 1–3 [file 6604688x1.ppt]

## Slide 1
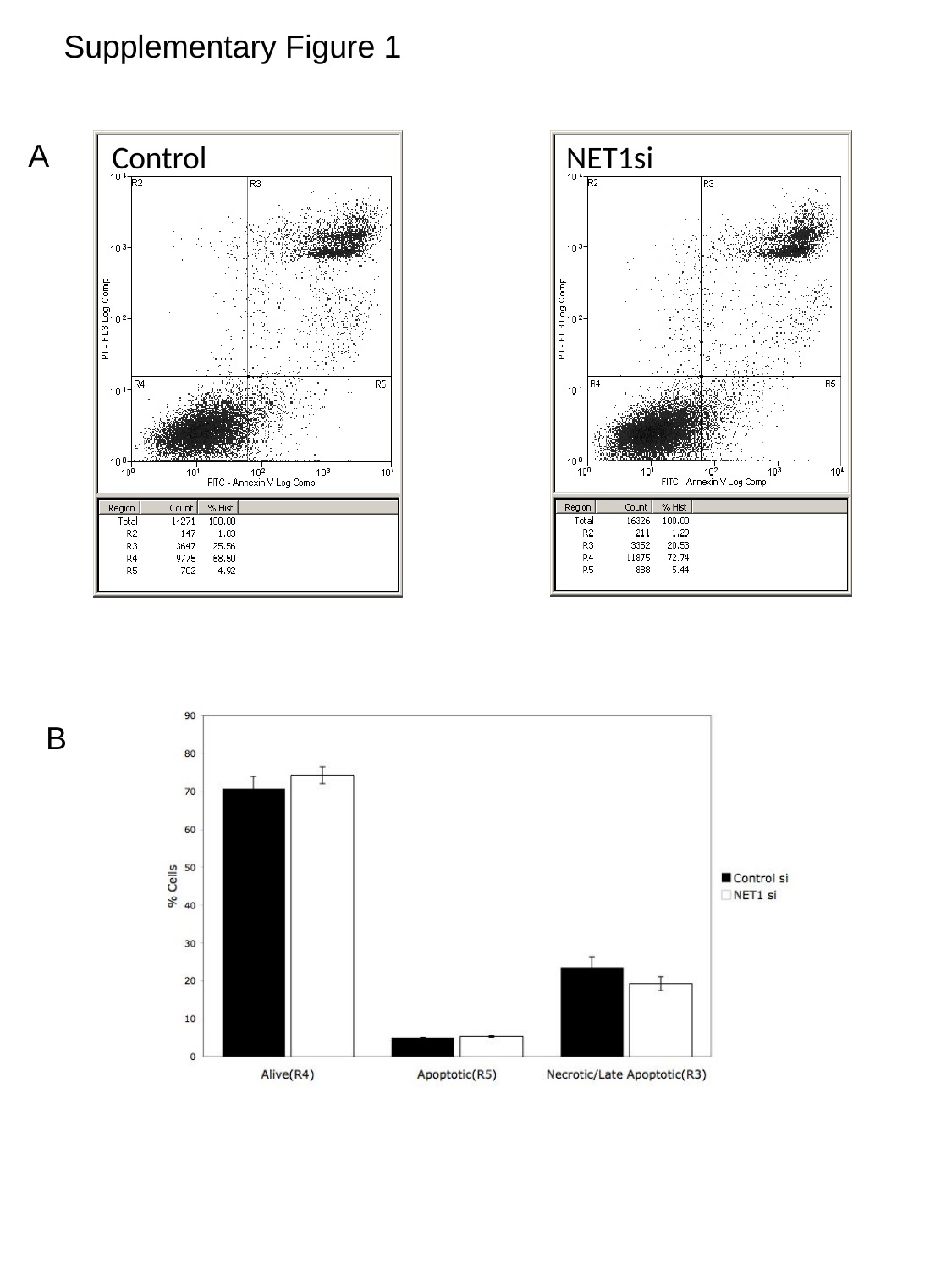

Supplementary Figure 1
A
Control
NET1si
B

## Slide 2
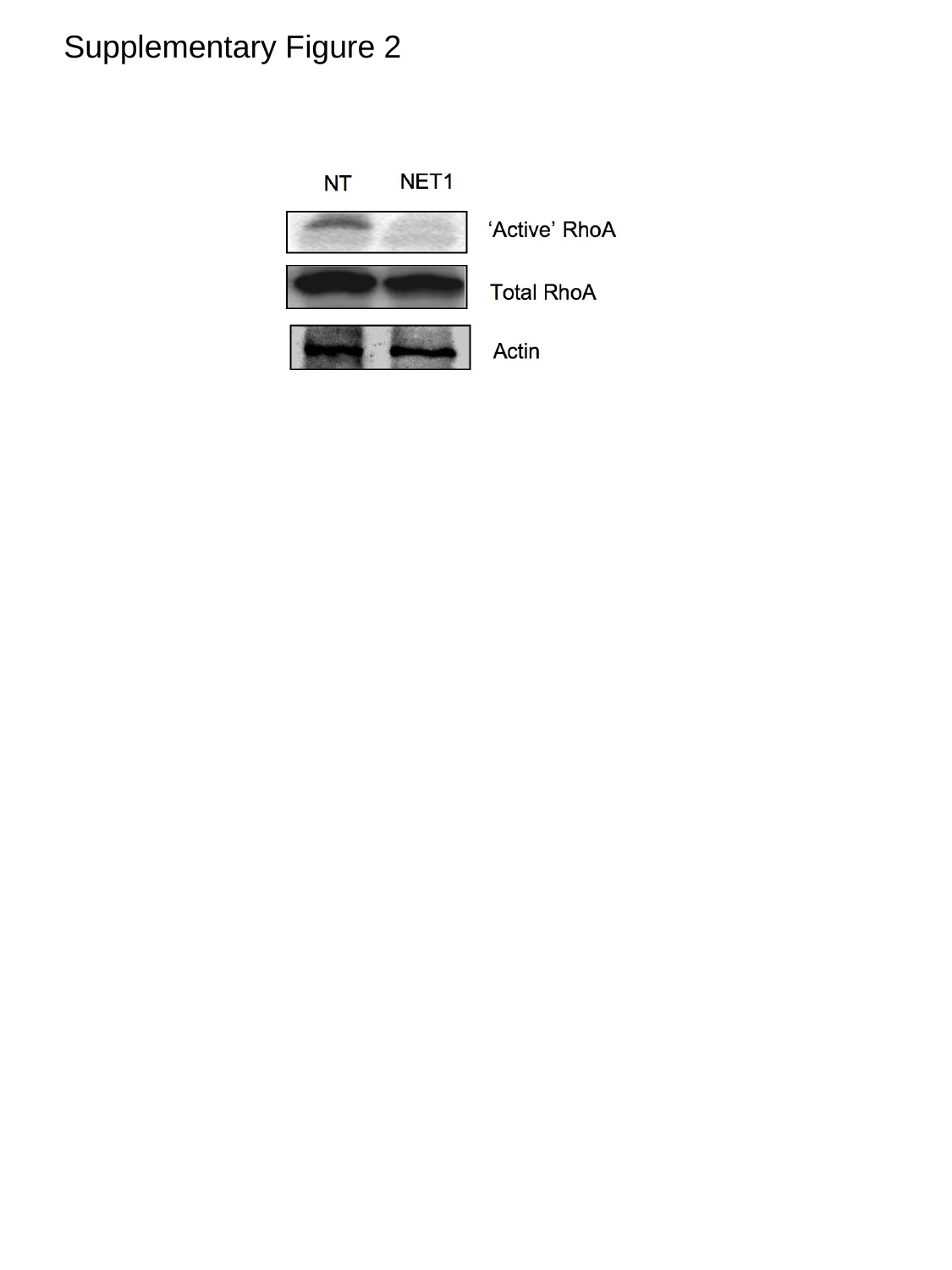

Supplementary Figure 2

## Slide 3
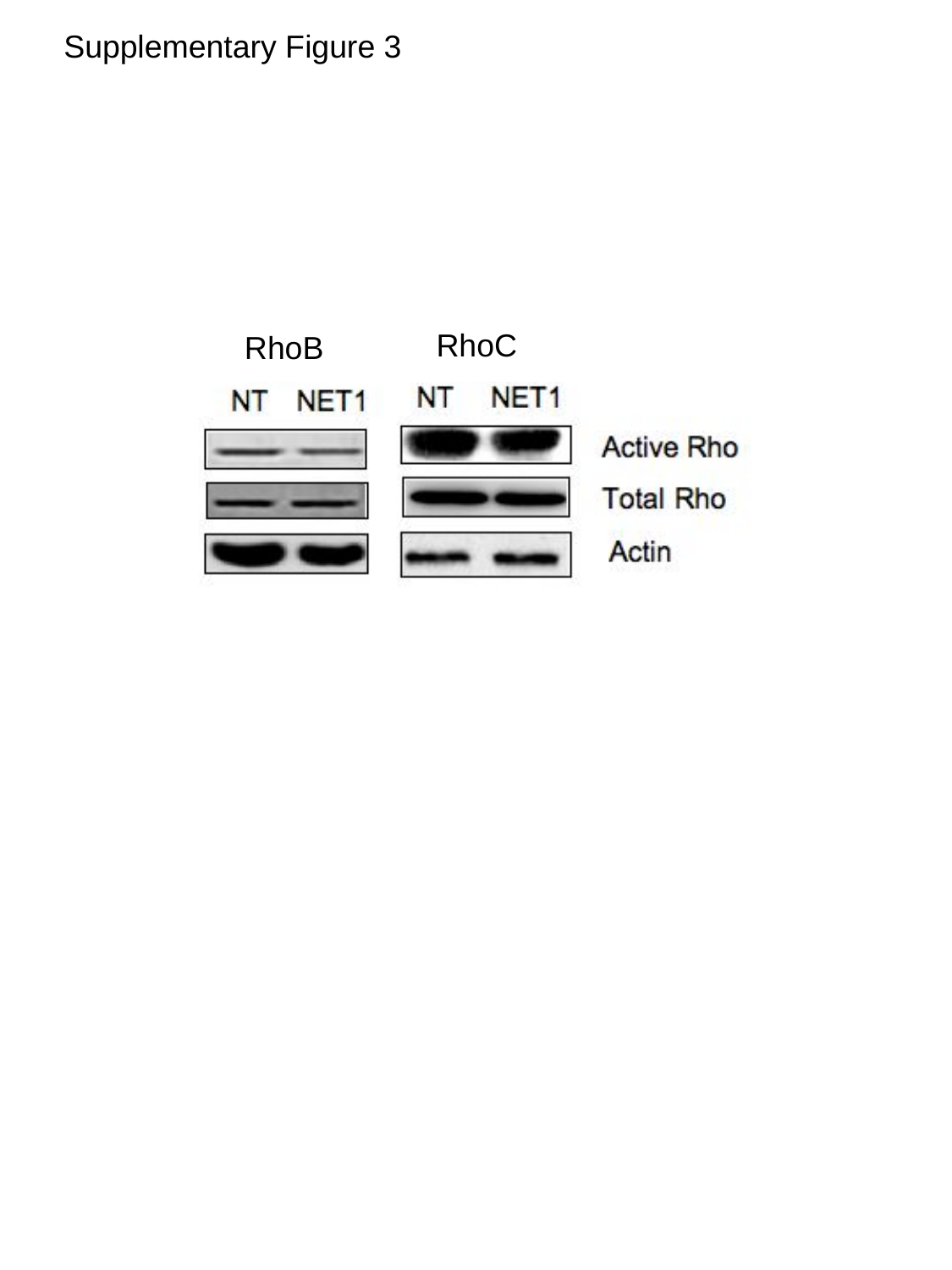

Supplementary Figure 3
RhoC
RhoB
