# Supplementary material for: NET1-mediated RhoA activation facilitates lysophosphatidic acid-induced cell migration and invasion in gastric cancer
Source: Br J Cancer. 2008 Sep 30;99(8):1322–9. doi: 10.1038/sj.bjc.6604688 (PMC2570507; doi:10.1038/sj.bjc.6604688)
Supplement: Supplementary Figures Legends [file 6604688x2.doc]

**Supplementary Figure 1.** *Assessment of cell viability in siRNA treated cells using flow cytometry.* A: Representative histograms of control siRNA and NET1 siRNA (75nM) treated cells. B : The % cells determined to be alive, undergoing apoptosis or necrosis.

**Supplementary Figure 2.** *Confirmation of NET1 mediated RhoA activation using a alternative siRNA duplex pair.* Western blot analysis of control and RNAi treated AGS cells. First and Second panel : ‘active’ and total RhoA respectively. Third panel : ß-actin (loading control). All western blot analysis was repeated in triplicate.

**Supplementary Figure 3**. Assessment of active and total RhoB and RhoC levels in siRNA-mediated NET1 knockdown cells and control non target (NT) cells. First and Second panel : ‘active’ and total Rho B and C respectively. Third panel : ß-actin (loading control). All western blot analysis was repeated in triplicate.
